# Effects of using mobile instant messaging on student behavioral, emotional, and cognitive engagement: a quasi-experimental study

**DOI:** 10.1186/s41239-021-00306-6

**Published:** 2022-01-11

**Authors:** Ying Tang, Khe Foon Hew

**Affiliations:** Faculty of Education, Southwest University, 2 Tiansheng Road, Beibei, Chongqing, China

**Keywords:** Mobile instant messaging, Asynchronous online discussion, Engagement, Higher education, Quasi-experimental

## Abstract

Mobile instant messaging (MIM) has become the most popular means for young people to communicate. MIM apps typically come with a myriad of features that educators may leverage to increase student learning. However, it remains poorly understood to what extent and in what aspect MIM can facilitate student engagement. We address the gap by comparing the effects of using MIM and asynchronous online discussion (AOD) on student online engagement, using a quasi-experimental study involving a historical cohort control group. Understanding which communication mode can better promote student online engagement is particularly important during the current widespread use of online learning. Specifically, we examined engagement from the behavioral, emotional, and cognitive dimensions. The results showed that the MIM group was more behaviorally engaged in discussion activities, producing more messages, more words, and higher rates of participation, task completion, and interaction. Emotionally, no statistically significant difference was found in students’ affective evaluation of course interaction and satisfaction between the two groups. However, MIM appeared to help students with improved intimacy and interpersonal relationships. Cognitively, the MIM group was more engaged than the AOD group. In particular, MIM seemed to facilitate interactive idea exchange and thus contributing to more “creating” activities. We conclude by providing suggestions for future instructional practice and research directions.

## Introduction

With the wide penetration of smartphones and mobile broadband access, mobile instant messaging (MIM) is becoming an essential means of communication worldwide (Dhir et al., 2020). As of July 2020, WhatsApp was the most popular MIM app with 2 billion monthly active users, followed by Facebook Messenger (1.3 billion) and WeChat (1.2 billion) (Statista, 2021). A recent study showed that MIM has surpassed voice calls, emails, face-to-face communication, and social network sites (e.g., Facebook, Twitter, Instagram) and become the most popular means of daily communication (Pew Research Center, 2019). Typical MIM apps offer various functions including group chats, audio/video chats, file sharing, real-time location sharing, and exchange of nonverbal graphics such as emoji and stickers.

**Fig. 1:**
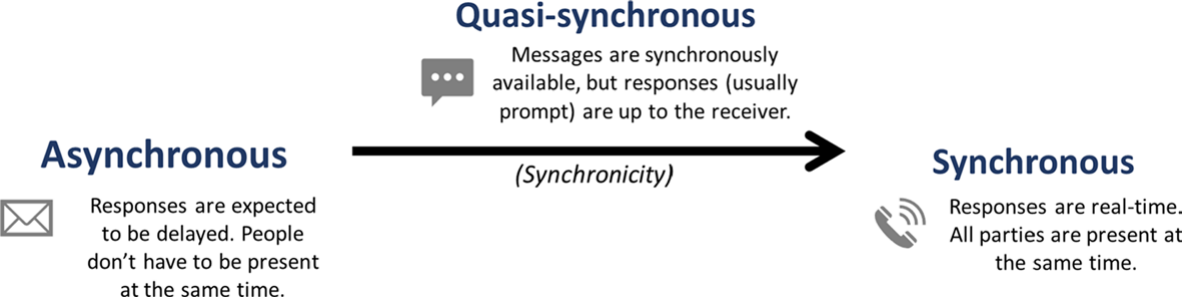
The differences between asynchronous, synchronous, and quasi-synchronous communication

MIM presents a unique “quasi-synchronous” communication because although posted messages are available synchronously to participants, the message production process (typing) is available only to the sender; the recipient does not have direct access to real-time message production by the sender (Garcia & Jacobs, 1999). When a new message arrives, a push notification will pop up and prompts users to engage in communication either instantaneously or with a short time lag. In other words, whether and when to participate in the communication is up to the message receiver after they get notified about message arrival. This is different from synchronous communication (e.g., phone calls, video chats), which requires the transmitter and receiver to be present at the same time and/or space at a mutually agreed schedule. There is no response time delay in synchronous communication because it happens in real time, unlike MIM quasi-synchronous communication where there is often some short delay. MIM is different from asynchronous communication (e.g., emails, forum discussions) because most MIM messages are answered promptly (Andujar, 2019) within 60 s (as in the case of WhatsApp) (Rosenfeld et al., 2018), whereas the average response time of asynchronous communication is markedly longer—24 h for email (Chang et al., 2016) and 24 to 48 h for online discussion forums (Jeong & Frazier, 2008). Figure 1 illustrates the differences between asynchronous, synchronous, and quasi-synchronous communication with examples.

MIM is both a mobile and quasi-synchronous communication tool. MIM is developed specifically for mobile devices and not for computers, which means users can carry WhatsApp or WeChat easily in their pocket (Unuth, 2020). The mobility of MIM tools and the quasi-synchronicity of MIM communication are two interwoven features and are not separable from each other. The quasi-synchronous communication of MIM is attributed to the portability and mobility of mobile devices. Thus, the word “quasi-synchronicity” has the connotation that easy accessibility of a mobile device is available in the communicative process. To explain, the message notification of a mobile phone alerts a receiver whenever a new WhatsApp or WeChat message arrives. The receiver can then choose to join the conversation anytime and anywhere he or she prefers.

In addition to its unprecedented popular social use, MIM demonstrates the potential to support teaching and learning. Like other computer-mediated communicative (CMC) channels, MIM shatters the temporal and spatial restrictions of traditional face-to-face meetings and allows people to stay connected (Kiesler et al., 1984). Besides, its multi-functionality and flexibility enable learning by facilitating resource sharing and distant collaboration (Tang & Hew, 2019; Xue & Churchill, 2019). For example, teachers have used MIM to support language practice (e.g., Andujar, 2016), after-class tutorial services (e.g., Butgereit, 2007), class-related information delivery (e.g., Chai & Fan, 2016), and assignment submission (e.g., Dambal et al., 2015). According to a recent literature review, MIM seemed to be particularly beneficial for developing a social presence in computer-mediated learning environments, mainly due to the friendly environment created with multiple integrated lively elements (e.g., visuals, audio, videos, and other graphical icons), as well as the increased interactivity of the quasi-synchronous communication (Tang & Hew, 2017). As for its impact on improving student learning outcomes, most experimental studies reported positive effect, especially when MIM was used to supplement course content in interactive class activities (e.g., Andujar, 2016; Chai & Fan, 2016). However, researchers also found that messaging can be obstructive to student learning, such as taking a longer time to complete the task or having lower assignments scores (Bowman et al., 2010; Chen & Yan, 2016; Fox et al., 2009). Unstructured messaging can also negatively influence one’s overall productivity due to the increase of communicative workload, engagement in multitasking, and frequency of message notification interruptions (Rennecker & Godwin, 2003).

Although the use of MIM has significantly increased, MIM has received much less attention in education, compared with other popular social tools such as Facebook and Twitter (Pimmer & Rambe, 2018). Questions remain concerning whether MIM is superior to other CMC mode (such as asynchronous online discussion [AOD]) in engaging students. In this study, we referred to the media synchronicity theory and examined the influence of MIM on learning through the lens of engagement, which is linked to desired learning behaviors and outcomes (Finn & Zimmer, 2012; Xu et al., 2020). We adopted a quasi-experimental research design to compare the influences of using MIM and AOD on student behavioral, emotional, and cognitive engagement. The central research question is: How effective is the impact of MIM-supported educational activities on student online engagement as compared to AOD?

This study offers the following original contributions. First, we empirically compare the extent to which MIM influences student online engagement with the commonly used AOD mode. Understanding which communication mode can better promote student online engagement is particularly important during the current health crisis. Since the outbreak of the COVID-19 pandemic, many institutions have little choice but to use online education for remote teaching and learning. Yet despite the widespread use of online education, the lack of student online engagement remains a problem (Farrell & Brunton, 2020). This study offers timely empirical evidence to help teachers choose the appropriate communication mode to foster student online engagement. Second, we investigate student engagement as a multi-dimensional construct, uncovering the nuances in how different communication modes influence student engagement behaviorally, emotionally, and cognitively. Third, we provide pedagogical suggestions to promote student engagement and learning in MIM-supported educational activities in CMC contexts.

In the following sections, we first review related literature on media synchronicity theory, the multidimensional nature of engagement, as well as empirical studies of the educational use of MIM. We proceed to describe the research design and present the comparative results of the behavioral, emotional, and cognitive engagement of participants from the two groups. We discuss the results in relation to media synchronicity and student motivation and conclude with a set of instructional design suggestions and directions for future research.

## Literature review

In this section, we review related literature on the media synchronicity theory, the multiple dimensions of engagement, as well as previous studies on the educational use of MIM to support student learning.

### Media synchronicity theory

Media Synchronicity Theory (MST) discusses the capability of media to support synchronicity, which is defined as “a state in which individuals are working together at the same time with a common focus” (Dennis et al., 2008, p. 581). According to MST, one medium is no better than another; communication performance can be improved when the synchronicity of media can match the synchronicity required to complete the task. Dennis et al. (2008) defined two fundamental processes of all communication: conveyance and convergence. Conveyance refers to the transmission of new information to create new mental models. Convergence is the process of reaching mutual understanding based on sharing “known” knowledge (Dennis et al., 2008). Since conveyance involves more cognitive processing of new information, it typically requires longer periods of time characterized by a medium with low synchronicity. In contrast, because convergence typically requires rapid transmission of small amounts of known information, it benefits from a medium that supports high synchronicity (Dennis et al., 2008). In real-life scenarios, to improve the performance of a communicative task, we need to consider the task nature, the media features, and the maturity of grouping, in order to make strategic choices of selecting and combining multiple media types (Dennis et al., 2008).

### Engagement as a multi-dimensional construct

**Fig. 2:**
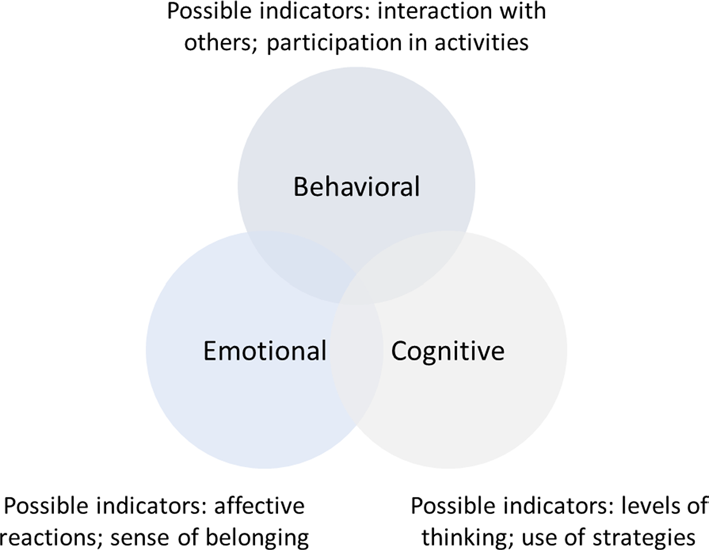
Three dimensions of engagement and examples of indicators for each dimension

Students engagement happens when they are motivated to devote time and efforts to the learning process (Wigfield et al., 2006). Engagement is the visible manifestation of motivation (Skinner et al., 2009). “Engagement is defined by an observable, action-oriented subtype (behavioral) and two internal ones (cognitive and emotional) but then is differentiated from motivation as engagement being action (observable behavior), motivation as intent (internal)” (Reschly & Christenson, 2012, p. 14). Engagement is multi-dimensional (Appleton et al., 2008; Finn & Zimmer, 2012). Fredricks et al. (2004) proposed a three-construct typology consisting of behavioral engagement, emotional engagement, and cognitive engagement (see Fig. 2, with two examples of indicative behaviors for each dimension).

This typology is well grounded in the literature and has been widely cited across diverse disciplines (e.g., Balfanz et al., 2007; Brodie et al., 2011; Hidi & Renninger, 2006). *Behavioral engagement* refers to participation in academic, social, or extracurricular activities (Fredricks et al., 2004). Student participation in an online discussion can be evaluated by various factors, such as the number and type of discussion posts (Hew & Cheung, 2003). *Emotional engagement* refers to students’ affective reactions toward interactions with teachers, peers, and the learning environment (Fredricks et al., 2004). Emotional engagement is typically measured by self-reported data, such as questionnaire surveys and interviews. *Cognitive engagement* comprises thinking and understanding of the topic, as reflected by students’ task investment in terms of being strategic or self-regulating (Fredricks et al., 2004). There is a qualitative distinction between low-level and high-level cognitive engagement, observable from surface-level to deep-level strategy use (Pintrich & De Groot, 1990). Cognitive engagement can be assessed either subjectively with self-reported questionnaire surveys or objectively with test scores and choices of task difficulty levels (Hew et al., 2016; Pintrich & De Groot, 1990). It is also common to perform content analysis to examine cognitive engagement in online interactions (e.g., Wang et al., 2014; Xie & Ke, 2011; Zhu, 2006).

### Education use of MIM

Previous studies have exploited the communicative functions of MIM and used it in dialogic activities. For example, Allagui (2014) asked students to accomplish structured conversation tasks (e.g., a role play) in WhatsApp groups to learn English. Over 80% of the students liked MIM and expressed willingness to continue using it. Similarly, Lai (2016) asked 45 seventh graders to use WhatsApp groups to practice English for three months and found a significant correlation between students’ chat frequency and vocabulary gain. Instructors also used MIM to deliver multi-modal messages or to provide out-of-class assistance in a timely manner. For example, Chai and Fan (2016) used WeChat to deliver texts, images, and videos about course content to support pre-class content delivery in a flipped classroom. Zhang and Xue (2015) allowed students to use WeChat and ask for help about their assignments or exam preparation. Aside from these two “transmission” and “helpline” functions, previous studies also used MIM to support other educational activities, such as to collaboratively complete a writing piece, to post a solution to an assignment, or to record students’ learning reflections (Tang & Hew, 2017). More recently, Xu et al. (2020) examined the effects of the teacher role on learner engagement in WeChat-based discussion.

MIM promotes a sense of collaboration among students (Robinson et al., 2015). Its quasi-synchronicity allows students to respond quickly, leading to an increased level of interactivity and the development of a social presence (Tu & McIsaac, 2002). Social presence refers to the ability of students “to project themselves socially and emotionally as real people” in mediated environment (Garrison et al., 1999, p. 94). It can influence student learning in the aspects of motivation, activity participation, course satisfaction, perceived learning, and critical thinking (Richardson et al., 2017). Furthermore, the availability of non-verbal cues, such as emoticons, emojis, and stickers, can convey emotions in student interactions (Tang & Hew, 2019) and make online conversations livelier and friendlier (Wang et al., 2016). Recently, Tang and Hew (2020) compared the levels of social presence between students using MIM and those using AOD and found that MIM is particularly suited to promoting expressions of emotions (affective social presence), agreement (interactive social presence), as well as phatics and support (cohesive social presence). However, this study focused solely on the social aspect of MIM use but did not examine its influence on other dimensions of students’ learning.

The effect of using MIM on cognitive engagement is not conclusive, due to the limited number of comparative studies and insufficient methodological rigor in experimental studies. For instance, Andujar (2016) and Chai and Fan (2016) reported positive effects of MIM use compared with the use of other tools or no treatment, while Kim et al. (2014) and Lai (2016) reported either no effect or adverse effects. A recent study by Sun et al. (2018) compared interaction types, social network patterns, and participants’ attitudes between using an online discussion forum and an MIM app. The results show students were more involved in social interactions on MIM but were more involved in knowledge construction on the online forum (Sun et al., 2018). While MIM led to more interactions, students preferred using the online forum for collaborative learning (Sun et al., 2018). This study adopted a broad categorization of interaction types (social interaction vs. knowledge construction) and did not evaluate specific levels of thinking or knowledge construction. Moreover, it only reported descriptive statistical results but no measurable learning outcomes.

The educational use of MIM also has challenges. Pimmer and Rambe (2018) identified three interdependent pairs of affordances and constraints in temporal, relationship, and intellectual dimensions. To explain, its immediacy may increase interactivity but may pressure users to respond quickly. Ubiquitous interaction may increase perceived intimacy but may decrease the sense of privacy. Informal language use may be considered friendly but may not be always appropriate in formal educational contexts. The casual environment may increase the level of playfulness and participation but may distract students from task-oriented conversations (Pimmer & Rambe, 2018). Other challenges include technical problems such as insufficient smartphone ownership, unstable Internet connectivity, and small cellphone keyboards and screens (e.g., Allagui, 2014; Dambal et al., 2015).

We identified three major gaps regarding student engagement and the educational use of MIM. First, there is a lack of empirical study that examined the influence of MIM-supported activities on student engagement. Second, some engagement indicators, such as student participation and affective responses, have been sporadically presented by previous studies. However, without a comprehensive examination of engagement as a multi-dimensional construct, our understanding of the educational potential of MIM remains limited. Third, very few studies have compared the effectiveness of using MIM with other communication mode in influencing student learning. This study addresses these gaps by comparing the impacts of using MIM and using AOD in educational activities on student engagement. In the next section, we present more details of the research design.

## Method

We conducted a quasi-experimental research involving a historical cohort control group. When random assignment of participants is not possible for practical and ethical reasons in educational research, a quasi-experimental study allows researchers to conduct comparative study in its natural setting (Campbell & Stanley, 1966). In addition, use of a historical cohort control group design provides a viable option for conducting quasi-experiments in outcome evaluation, with minimal resource requirements and disruption to school routines (Walser, 2014). To manage the comparability of treatment and control conditions, in this study, we chose two classes taught by the same instructor with identical syllabi, course materials, and class activities in two consecutive semesters. Students in the experimental group (hereinafter: MIM group) used WeChat for course-related discussion, while those in the control group (hereinafter: AOD group) used the asynchronous Moodle forum for the same activities. Moodle is the learning management system used on the university campus.

This study was conducted in an ecologically valid real classroom setting where WeChat and Moodle were adopted in natural educational practices, instead of a laboratory setting where strict controls of experiment conditions to investigate the different influences of mobile versus non-mobile, and synchronous versus asynchronous communication were imposed. In this study, we did not impose any restrictions on how participants should use MIM or AOD services. All students in the MIM group naturally used the MIM service via the mobile app on their mobile phones while all students from the AOD group naturally used Moodle forum via their personal computers. This allowed us to better understand what was going on in real classrooms and derive insights from authentic use cases. Moreover, although we chose these two tools for this study, our focus was not to merely compare these tools, but to evaluate the impacts of different modes (quasi-synchronous communication and mobility presented by MIM versus asynchronous communication and non-mobility represented by Moodle forum) on student engagement. MIM and AOD forum are both widely adopted to support student learning activities. Understanding which communication mode can better promote student online engagement is particularly important during the current widespread use of online learning. Our goal was to empirically investigate which one of the communicative modes can better support student engagement in real classroom settings.

To further improve the validity of comparison, since students with a better understanding of the content knowledge typically participate more actively (Tinto, 1987), we controlled the influence of students’ initial content knowledge by administering a pre-class assessment on the main knowledge unit covered in this course. 26 students from the MIM group and 28 from the AOD group completed the quiz. Since the data significantly deviated from a normal distribution, we compared the difference with a Mann–Whitney *U*-test. The results showed no significant difference (*U* = 337.5, *p* = 0.63) in student prior knowledge between the two groups (MIM group: *M* = 1.19, *SD* = 1.44; AOD group: *M* = 1.21, *SD* = 1.13) (See Table 1).

**Table 1:** Comparison of pre-class quiz scores between the two groups

Group	Number of participants	M (SD)	Mann–Whitney U	Wilcoxon W	Z	Asymp. Sig. (2-tailed)
MIM	26	1.19 (1.44)	337.5	688.5	− 0.48	0.63
AOD	28	1.21 (1.13)				

### Research context

**Fig. 3:**
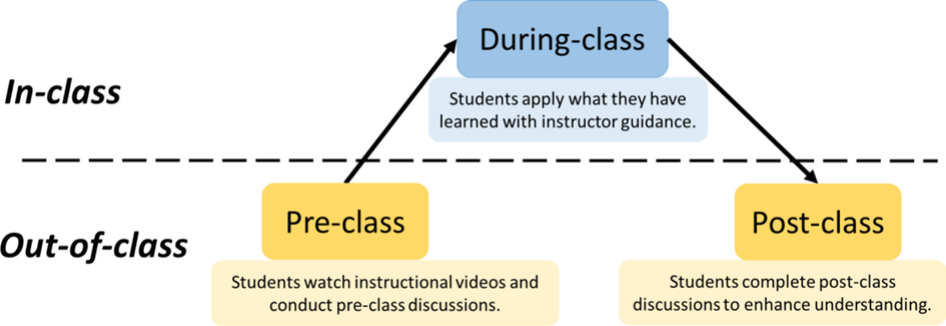
The learning activities and process of a flipped learning approach adopted in this study

Participants were enrolled in an educational course at a large university in Hong Kong in the 2016–2017 school year. The class met once a week, three hours each week, and for eight weeks in total. The instructor taught the course in a flipped approach, by disseminating instructional videos before each class and guiding students to apply what they had learned in class (see Fig. 3 for a visual illustration of the learning process). The first four weeks covered content knowledge, and the last four weeks were for student-led group presentations. In the first four weeks, students completed six online discussion tasks (see Table 2) on the designated platforms. The MIM group was enrolled in 2016 Fall, involving 26 students (23 females and three males). All the students were from mainland China or Hong Kong. The AOD group was enrolled in 2017 Spring, involving 29 students (21 females and eight males). One student was from Thailand, and the others were from mainland China or Hong Kong.

**Table 2:** Session topics of the first four weeks and six discussion tasks

Week	Session topic	Discussion task
1	Adult learners	*Post-class:* Introduce one adult class you taught before or wish to teach
2	Andragogy	*Pre-class:* Watch a video and propose two solutions to address the situation
		*Post-class:* Discuss one topic from last class with which you feel most connected
3	Motivation	*Pre-class:* Talk about possible methods of positive reinforcement, negative reinforcement, and punishment
		*Post-class:* Read the article and post one concept you find most interesting
4	Online learning	*Pre-class:* What are three most important questions to consider about designing an online course for adults? Why?

Students understood that their participation in the discussion was completely voluntary and would not be counted toward their grades. Students were encouraged to provide feedback to others’ comments, and they were allowed to use the discussion platform in any way that might help them learn with no prescribed regulations. For example, they could ask assignment-related questions or share internship information. The instructor did not participate unless students specifically sought his help.

### Data collection and analysis

**Fig. 4:**
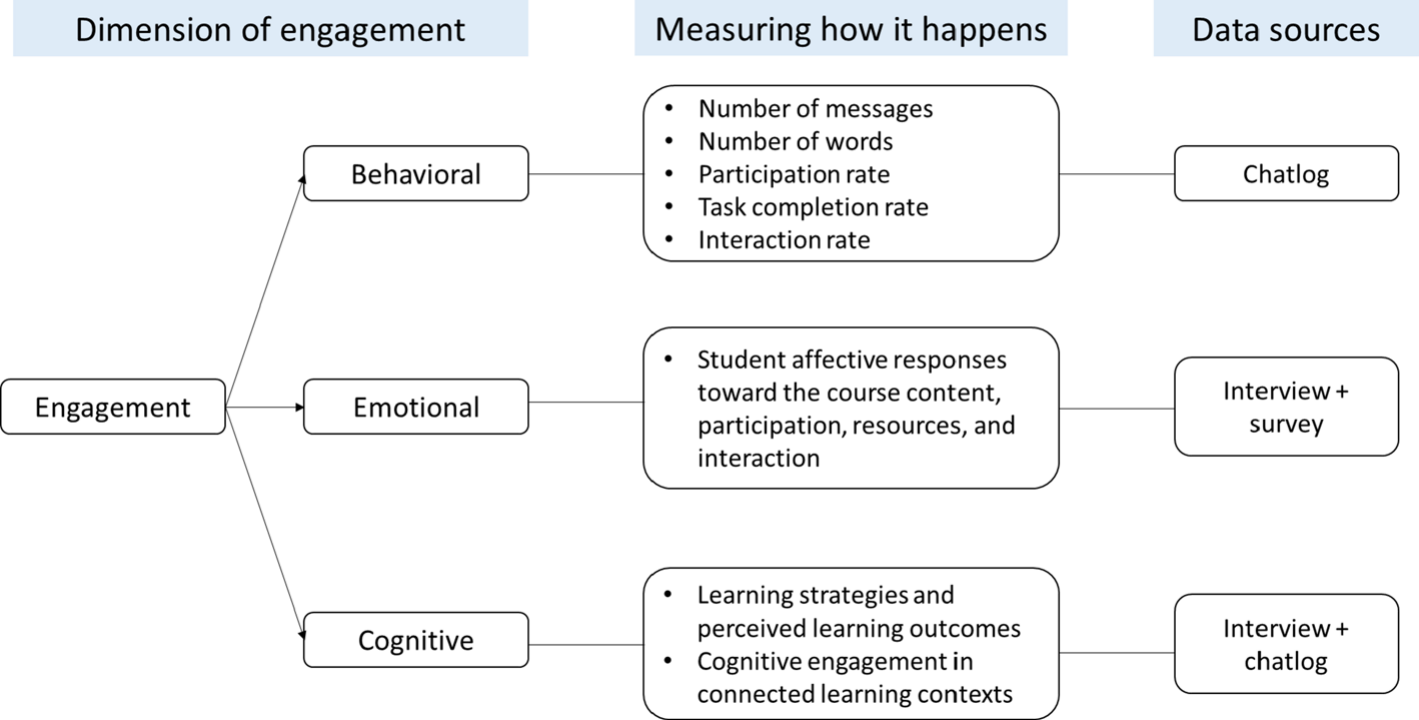
The measurements and data sources of behavioral, emotional, and cognitive engagement

Figure 4 summarizes the ways in which we measured how behavioral, emotional, and cognitive engagement happened in the learning process, and how we collected the data.

To measure *behavioral engagement*, we collected students’ online interaction records after the course ended, and analyzed the data using a content analysis method (Holsti, 1969). Specifically, we counted the number of participants and their posts, as well as calculated the number of posts per individual and discussion task. We categorized postings as on-task and off-task messages. On-task messages were posts that directly contributed to the discussion topics, while off-task messages were not directly related to the topics. On-task messages were further grouped into two types: task completion (messages intended to complete the task) and interaction (messages as responses). One message could contain components of both task completion and interaction.

To compare *emotional engagement*, we interviewed 20 students, 10 from each group, to understand their affective responses toward the course content, participation, resources, and interaction. These interviews were semi-structured, wherein we asked elaboration and clarification questions as needed to gain more insight. Each interview lasted about 30 min. All interviews were audio recorded, transcribed verbatim, and double-checked for accuracy. We also administered a Likert-style survey anonymously via Google Forms upon the course completion. The 15-item survey was adapted from Bailey (2002) to measure student attitude towards peer interaction, student–instructor interaction, and course satisfaction. We compared students’ responses with the Mann–Whitney *U*-test, as the data significantly deviated from a normal distribution. Significance was accepted at the level of 0.05.

The aforementioned interviews also helped identify students’ learning strategies and perceived learning outcomes, as indicators of *cognitive engagement*. We analyzed the interview data with a grounded theory approach (Glaser & Strauss, 1967) to allow categories to emerge from the data. This involved generating a series of codes and successively refining them through an iterative process, until no more new codes could be identified.

In addition to the interview data, we adopted Wang et al. (2014) framework of cognitive engagement in connected learning contexts and analyzed on-task messages as an externalization of learning-related thought. The unit of analysis was the thematic unit because our focus was the “communication of meaning” (Merriam, 1998). One message might contain several units of analysis. The framework includes four levels (from low to high) of cognitive engagement:

1. **Operation**. Learners operate technologies to help with their personal learning and mainly interact with the interface to facilitate idea expression. This stage does not involve interaction with other participants.
2. **Wayfinding**. Learners identify resources and opportunities contributed by others in the network. Learners interact with the content and other learners to aggregate more information to enhance their understanding.
3. **Sense-making.** Learners bring together different information and critically evaluate and negotiate a viewpoint.
4. **Innovation.** Learners construct new understanding and artifacts, such as changes to one’s personal opinions or behaviors and new suggestions or resources to extend the existing discussion.

Guided by this framework, we adopted the constant comparison method (Lincoln & Guba, 1985) to identify specific indicators of cognitive engagement. Ten codes emerged inductively from the data corpus. The operationalization of each code was reviewed and refined iteratively. To increase the consistency of the analysis, we identified exemplary posts to illustrate each code. The final codebook is shown in Table 3. Twenty percent of the qualitative data were randomly selected and analyzed by two independent researchers to ascertain coding reliability, which yielded an agreement rate of 92%. All disagreement was resolved through discussion.

**Table 3:** Analytical framework of cognitive engagement

Code	Definition	Example
Operation		
Sharing information	Providing an opinion or information	“I will prepare more videos to share with the class when things get boring.”
Seeking information	Proposing of an original question	“The speaker only shared the first two points. Does anyone know the last three points?”
Wayfinding		
Commenting without elaboration	Commenting on others’ ideas without elaboration	“Your sharing is very interesting!”
Requesting elaboration	Requesting for more information or inviting discussion	“I am wondering if there [are] any good examples to teach different levels of students.”
Providing elaboration	Adding explanation or justification of one’s own ideas	(After proposing a solution). “If adults are feeling sleepy in class, they must be really tired.”
Summarizing	Summarizing knowledge with little evaluation	“I agree with my previous classmates’ ideas, such as instant Q&A from A; separate tasks from B, and a change in topics from C.”
Sense-making		
Analyzing	Analyzing essential features, comparison, and reasons	“The discrepancy between staff needs and training requests was a key factor!”
Evaluating	Stating a stance with justification	“Your suggestion is inspiring! Giving students more opportunities to talk could change passive listening into active participation!”
Innovation		
Reflecting	Reflecting on one’s prior experience or learning outcomes	“A big mistake I made was that I started with pronunciation and tried to teach from A to Z.”
Creating	Creating new ideas by making suggestions, extending understanding, and introducing new points or resources	“You may initiate e-learning in a less formal setting such as a tutorial class and give teachers detailed examples of how e-learning works.”

## Results

### Behavioral engagement

Table 4 summarizes the differences in behavioral engagement of the two groups.

**Table 4:** Differences in behavioral engagement

Measurements	MIM	AOD
Number of students enrolled	26	29
Number of students who participated in discussions	26	28
Number of messages	473	169
On-task messages	321	169
Interaction messages	186	47
Number of words in total	22,489	17,289
Participation rate	88.5%	69.5%
Task completion rate	81.4%	69.5%
Interaction rate	51.7%	23.1%

The MIM group produced more messages than the AOD group, including more on-task and interactive messages, while the AOD group wrote longer messages than MIM students. All MIM students participated in the out-of-class discussions, producing 473 messages with 22,489 words. A total of 321 messages were on-task, among which 186 (57.9%) were interactive. Each student produced 865 words, and each message contained an average of 47.5 words. On the other hand, 28 of the 29 students from the AOD group participated in the tasks, producing 169 messages with 17,289 words. All the messages were on-task, and 47 (27.8%) were interactive. The average number of words per student was 617.5 and per message was 102. We further analyzed the following aspects of participation and interaction.

#### Participation rate

Measured by dividing the number of students who participated by the total number of students enrolled. The average participation rate of the MIM group across all six tasks was 88.5% and that of the AOD group was 69.5%. The MIM group demonstrated a higher participation rate.

#### Task completion rate

Measured by dividing the number of students who completed the tasks by the total number of students enrolled. In the MIM group, the average number of students who completed the tasks was 21.2, with a completion rate of 81.4%. In the AOD group, the average number of students who completed the tasks was 20.2, with a completion rate of 69.5%. The MIM students demonstrated a higher task completion rate.

#### Interaction rate

Measured by dividing the number of interaction posts by the total number of posts. The MIM group’s interaction rate reached 51.68%, while the AOD group’s interaction rate was 23.13%. In the AOD group, two tasks did not have any interactive posts. The MIM students demonstrated a higher interaction rate.

### Emotional engagement

According to the interviews, both MIM and AOD helped students create a sense of group and connectivity. Both technologies improved an awareness of peer presence. However, while most MIM interviewees explicitly acknowledged the positive impact of using WeChat on their feelings, hardly any AOD interviewees recognized the effect of the forum on their emotional engagement. Students mentioned the following reasons why they felt WeChat enhanced their emotional engagement.

#### Instructor being approachable

Students appreciated the instructor’s attempt to use this social tool in an academic context. According to one interviewee, “It shows he is willing to know us and mingle with us. I do not know any other teachers who are using WeChat. Maybe they do, but they don’t share it with us.”

#### Inclusive, relaxing, and interactive

Students frequently used inclusive pronouns, such as “we, us, our” to address the group, which gave them a sense of belonging. In addition, the message notifications prompted them to get engaged and contributed to an interactive learning environment. One student noted, “You know others are participating, and you want to be a part of it too. It is not like Moodle, where you post your answer and leave. I seldom read what others say, and I don’t think others care that much about what I say.”

#### Easy emotional expression

The convenient use of emojis and stickers helped with emotional expression and added some fun to the conversation. One student said, “If someone knows how to use stickers, they are more likely to be an easygoing and interesting person. I would want to know them and make friends with them.”

#### Social interaction

The social nature of WeChat increased a sense of intimacy. Students would use social phatics, such as greetings or holiday wishes, to improve the level of positivity and friendliness. Students were also connected with the social sharing function, which allowed them to know their classmates as unique individuals outside of the classroom. One student commented, “You get to know them better through what they share and how they describe their posts.”

However, some students still preferred limiting the use of MIM to social conversations. They felt awkward to intentionally use MIM for academic posts. Further, students sometimes thought the notifications pushed by MIM were distracting, even annoying. Messages could also be obstructive to one’s private life. Students also disliked the pressure to participate only because their classmates were active, even though the discussions were voluntary. One student said, “Sometimes I forced myself to comment, because others would have a better impression of me, including the teacher.”

On the other hand, most AOD interviewees showed a neutral attitude toward the impact of using forum on their affective involvement. Their responses were comparatively succinct, such as “I could not say it had a strong influence,” or “It was not obvious.” Two main uninviting features of Moodle forum were (1) the lack of interaction, and (2) the pressure of being formal. Most students would just post their own answers but did not bother to comment on others’ postings. When they wrote their own responses, students felt the pressure to provide in-depth responses. One student said, “I always wrote an essay in a Word document, double-checked the grammar, and read it several times before posting it. I had to make sure that everything was up to academic expectations.”

Regarding the survey results, 20 MIM students and 27 AOD students responded on their attitudes towards the course interaction and satisfaction. The score of each construct was obtained by summing the scores of each item in that construct, and the overall score was obtained by adding the scores of all constructs. The results showed no significant difference in any surveyed aspects (see Table 5).

**Table 5:** Survey results of course interaction and satisfaction and comparison between two groups

Group	M (SD)	Mdn	Mean rank	Min	Max	Statistical test results			
						Mann–Whitney U	Wilcoxon W	Z	Asymp. Sig. (2-tailed)
Students’ peer interaction									
MIM	20.9 (4.3)	21.5	22.0	7.0	25.0	229.500	439.500	− 0.890	0.374
AOD	22.0 (3.5)	22.0	25.5	10.0	25.0				
Student–teacher interaction									
MIM	20.9 (4.3)	21.0	20.6	6.0	25.0	201.500	411.500	− 1.502	0.133
AOD	22.1 (3.5)	22.0	26.5	10.0	25.0				
Course satisfaction									
MIM	21.0 (4.0)	21.0	20.7	8.0	25.0	201.000	411.000	− 1.518	0.129
AOD	22.3 (3.5)	23.0	26.6	10.0	25.0				
Overall evaluation									
MIM	62.8 (11.8)	64.0	20.6	21.00	75.0	202.500	412.500	− 1.463	0.143
AOD	66.4 (10.2)	69.0	26.5	30.00	75.0				

### Cognitive engagement

We measured cognitive engagement based on students’ online interaction records and interviews about their learning strategies and perceived learning outcomes. Table 6 summarizes the coding results of students’ interaction records.

**Table 6:** Differences in cognitive engagement reflected in students’ posts

Dimension	Code	MIM	AOD
Operation	Sharing information	83 (24%)	76 (41%)
	Seeking information	3 (1%)	0
	Subtotal	86 (25%)	76 (41%)
Wayfinding	Commenting without elaboration	47 (13%)	3 (2%)
	Requesting elaboration	20 (6%)	9 (5%)
	Providing elaboration	32 (9%)	3 (2%)
	Summarizing	16 (5%)	6 (3%)
	Subtotal	115 (33%)	21 (12%)
Sense-making	Analyzing	19 (5%)	5 (3%)
	Evaluating	35 (10%)	27 (15%)
	Subtotal	54 (15%)	32 (18%)
Innovation	Reflecting	65 (18%)	47 (26%)
	Creating	32 (9%)	8 (4%)
	Subtotal	97 (27%)	55 (30%)
	Total	352 (100%)	184 (100%)

Both technologies supported student cognitive engagement in a variety of ways. A stark difference was in the wayfinding dimension. MIM students were most involved in wayfinding interaction, while the AOD students showed the least involvement in this category. This indicates MIM was more facilitative to interaction, as wayfinding indicates bidirectional communication. Regarding specific indicators, both groups were more frequently engaged in sharing information, although the AOD group demonstrated a higher percentage. Between groups, MIM students demonstrated higher frequencies of all individual indicators, indicating that more cognitively engaging communication was present in the MIM group. Particularly, MIM students demonstrated more instances of the highest level of cognitive engagement indicator “creating” than their AOD peers (32 vs. 8).

According to the interviews, students in the MIM group attributed their improved cognitive engagement to increased interactivity, class preparation, just-in-time learning opportunities, connected learning resources, and succinct language use.

#### Learning facilitated by increased interactivity

The mobility and synchronicity of MIM increased the level of interactivity and allowed students to seize just-in-time learning opportunities. They were able to co-produce knowledge in peer interactions, which prompted them to think more deeply about the discussion topics. One student said, “I would have more opportunities to ask questions and receive answers from my classmates. Reading my classmates’ answers also helped me learn.”

#### Better class preparation

Students actively utilized the MIM group to prepare for the course. “We basically explored the course content together before class. Unsolved questions could then be addressed by the instructor in class.”

#### Connected learning resources

The MIM group established an easy conversation channel, whereby they could navigate learning resources and have direct contact with their classmates to ask for clarification and elaboration. “If I am interested in a particular idea and would love to have more discussions, I could just @ the sender and ask them directly. It is convenient and efficient.”

However, some students from the MIM group complained about the size of keyboard and screen, which limited them to fully elaborate on ideas. Another negative factor was the chronological display of messages, which made group conversations difficult to follow. Students found it hard to engage in in-depth discussions if they were always catching up on the most recent messages. “If you are not following the interactions all the time, there is no easy way to read all the messages.”

As for AOD, students appreciated using it for academic discussions, mainly due to the following factors.

#### Extended processing time

Students took advantage of the asynchronous feature of the online forum to really think about their ideas and carefully put them into words. Extra processing time helped develop higher-order thinking, as one student said, “I can take my time to think carefully about what I want to say and how to support my ideas. I might spend several days on the draft.”

#### Searching while writing

All students used the online forum on their personal computers. The ease of searching and writing at the same time was one outstanding affordance of using Moodle forum. This feature was particularly facilitative of idea development, especially as academic posting tended to be complex and often required searching for extra information.

#### Easy editing and revision

It was easy to refine the content and insert more information on the forum. This allows for more critical thinking and language processing. “I can revise my posting when I have new ideas or supporting materials. It’s nice that Moodle allows editing.”

#### Organized threaded format

The threaded format of forum posts allowed students to organize relevant posts and easily search for content. Students considered this feature helpful for information retrieval and idea development.

What students disliked about AOD are its low interactivity and long formal responses. Like many other learning management systems, Moodle forum was not mobile-friendly, as it required students to take multiple steps to login, navigate to the right course, find the discussion board and the right topic, and then write their responses. One student said, “It is hard to use. That’s why I did not interact much. It was a headache to find the right place.” Additionally, students did not enjoy reading long posts or providing responses to such posts. The low interactivity and difficulty of use prevented students from actively participating in discussion tasks and thus limited the development of high-level cognitive engagement.

## Discussion and implications

### Revisiting the major findings

In this study, we compared the engagement levels of two groups of students using MIM and AOD to conduct same course-related discussions in a flipped learning setting. Behaviorally, MIM seems to have contributed to a higher level of engagement than AOD in terms of the total number of posts, the total number of words and of each student, the participation rate, the task completion rate, and the interaction rate. However, the messages tended to be longer on the forum than those in the MIM group, as shown by the number of words per message.

Emotionally, although the survey results showed no significant differences in student peer interaction, teacher–student interaction, or course satisfaction between the two groups, the interview results revealed more nuance. MIM established a friendly and interactive environment, which helped develop positive interpersonal relationships among participants. In contrast, students found little impact of AOD communication on their affective feelings toward the course and other students. On the negative side, some students disliked using WeChat for academic purposes, and some from the AOD group did not like the lack of interaction and lengthy essay-like responses on Moodle forum.

Cognitively, both technologies supported student cognitive engagement. AOD seemed more facilitative of individual sharing, while MIM contributed to a higher level of interactive idea exchange. The MIM group was also involved in more “creating” activities. Based on the interview results, students identified multiple facilitative and inhibitive features of both technologies. The MIM students enjoyed the increased interactivity, better class preparation, just-in-time learning opportunities, connected learning resources, and succinct language use, but they were also bothered by the device limitations and information disorganization. In the AOD group, students liked the structured discussion, extended processing time, and the ease of multi-tasking and editing, but they did not enjoy reading or responding to lengthy posts, which limited their desire for interaction.

The mobility, quasi-synchronicity, and casualness of MIM reduced students’ anxiety about being perfect and promote spontaneous discussion (Rambe & Bere, 2013). Studies have suggested that the increased interactivity would lead to a higher level of intimacy (Tu & McIsaac, 2002), which is a key factor of student affective feelings toward and satisfaction with completely online learning (Gunawardena & Zittle, 1997). However, our survey results revealed no significant difference in the emotional engagement between the two groups, although the MIM group demonstrated a higher level of interactivity. This might be due to the course nature, which in this study operated in a blended format. Therefore, the results may be different from completely online courses examined in previous studies. This course prioritized face-to-face meetings and included online discussions as supplementary and voluntary activities. As a result, online interaction might have less impact on student affective engagement than face-to-face meetings. Because both groups took the course with the same instructor and the same activities, their evaluation of the course might be very similar.

The differences in cognitive engagement presented a complex picture. Previous studies found that threaded AODs might better support higher-order thinking compared with chronologically organized discussions in instant messages (Kim et al., 2014; Sun et al., 2018). Our results suggested otherwise. One explanation of this incongruity may be that in both Kim et al. (2014) and Sun et al. (2018), the instructors assigned discussion activities as *mandatory* tasks. These activities were designed purposefully as collaborative projects, in which students had to either collaboratively find solutions to an ill-structured problem or co-develop a lesson plan based on peer feedback. According to the media synchronicity theory, when a large amount of information is to be exchanged in the communication, media that afford low synchronicity (AOD in this case) may facilitate better communication performance, as it allows more time to read, understand, and process information transmitted (Dennis et al., 2008). However, in our study, participating in the discussions was voluntary, and collaboration was not necessary. When students were given the choice to participate, MIM seemed to have afforded higher levels of interaction and more opportunities to share ideas. Such frequent “give and take” is favorably supported by a medium that supports high synchronicity (in this case, MIM) (Dennis et al., 2008). The increased level of interaction may have contributed to higher cognitive engagement, especially more “creating” indicators. The creation of new ideas or artifacts does not occur in an individual but through interaction between the individual and the social–cultural context (Csikszentmihályi, 1990). Pi et al. (2019) also found if students were exposed to a high rate of peers’ original ideas and paid more attention to those ideas, they would be more creative. Therefore, using MIM to boost students’ interaction might be a useful strategy for improving creative thinking.

### Instructional design suggestions

According to the media synchronicity theory, no medium is better than the other. Communicators should choose appropriate media based on the task requirements, the media features, and the maturity of grouping (Dennis et al., 2008). Based on our observation and analysis, we provide the following suggestions for instructors who wish to incorporate MIM or AOD to improve student engagement and learning. These suggestions aim to utilize the affordances of MIM or AOD and to address their challenges.

#### Set clear goals for using technologies for learning

Different communicative modes and tools are suitable to serve different learning purposes. To address students’ complaints over the confusion between casual and academic interactions, instructors should clearly communicate the purposes and expectations of using MIM or AOD for communication and learning activities. Instructor can also encourage students to design and implement MIM-supported learning activities themselves. Giving students the freedom of choice will enhance their sense of autonomy, leading to higher levels of behavioral and emotional engagement. Our study also shows that clear communication of the expectations will help students to be more intentionally focused of academic conversations.

#### Start with the whole-class MIM group first, but reconsider group size in discussion activities

The MIM group helps students to stay connected, assist one another with content learning, and exchange ideas to facilitate higher-level thinking. It is therefore beneficial to have a group to improve relatedness and competence. However, as indicated by some participants in this study, too many members in a group will lead to information overload and message disorganization. We thus suggest starting a whole-class group first to establish a sense of community but breaking up into smaller groups for discussion activities. For example, students could have internal discussion within small groups. Each group then selects a representative to summarize and present group ideas to the whole class to further facilitate exchange of thoughts.

#### Develop schedules for discussion

Instructor can develop a schedule with students to address student concerns about messaging being distractions and invasion of privacy, but still maintain flexibility and connectivity (Tu et al., 2014). For example, a discussion schedule can be set between 9 am and 5 pm each weekday. The schedule should be a collective decision of all participants. In addition, instructors can remind students to mute their MIM notifications when they do not want to be disturbed (Tang & Hew, 2019). AOD, on the other hand, due to its asynchronous nature, does not create any distraction or obtrusive feelings.

#### Design activities catering to device limitations

Students in this study complained about the low input ability and readability of long messages on MIM apps. They did not enjoy reading long messages on their cellphone screens and had difficulty typing long messages with small cellphone keyboards. Therefore, discussion activities should be designed purposefully with comparatively short answers that are easy to summarize. A task with multiple questions, or a topic requiring students to share anecdotal experiences in extensive narration, may be more suitable for AOD-based communication.

#### Cultivate a constructive knowledge sharing environment

High level of interaction is beneficial for enhancing both competence and relatedness. Instructors should actively cultivate a constructive knowledge exchange environment in MIM-supported interactions. For example, instructors can demonstrate how to provide constructive comments and build on one another’s ideas. To address information disorganization of MIM communication, in addition to managing group size, instructors could also assign student facilitators to summarize the highlights and manage conversations, which could help students to grasp crucial information and facilitate follow-up interactions. As for AOD, because of its asynchronous nature and comparatively low interactivity, instructors could consider providing rewards, incentives, or other positive stimuli to encourage students to participate in the knowledge co-construction process through interactions, in addition to their own sharing.

## Limitations and future work

There are some limitations of this study which point to an array of possibilities for future research. First, the study was conducted in a graduate-level education course. These contextual factors might limit the generalizability of our results to other contexts such as undergraduate and K-12 settings. Future studies could investigate other disciplinary and geographical areas and in non-higher educational contexts. Second, the study examined student use of one technology over one semester (eight weeks). There might be a novelty effect on the results—the tendency of an initial improvement in student performance when a new technology is introduced to the learning environment (Clark, 1983). Researchers might wish to examine the long-term adoption of MIM and its influence on student learning and engagement. Third, this study suggests that discussion tasks with shorter answers could cater to the strengths of mobile devices. However, it is beyond our scope to investigate what types of activities are most suited to an MIM-based discussion. Future research could explore the influence of different discussion activities. Fourth, this study does not demonstrate that how different levels of engagement eventually lead to qualities of learning outcomes. Further studies should examine the relationship between student engagement and learning outcomes. Finally, we were unable to isolate the possible individual influences of mobility and synchronicity on the reported results. To determine the possible individual influences of mobility and synchronicity, future research may consider having different experiment conditions while controlling for the confounding variable. For instance, one group may use quasi-synchronous communication (MIM) via mobile phones versus another group using asynchronous online discussion also via mobile phones.

## Conclusion

Our main motivation for this study is to understand the extent to which MIM, as a social tool, can facilitate student engagement. We compared the use and effects of MIM and AOD on students’ behavioral, emotional, and cognitive engagement. Our findings showed that using MIM could better facilitate interaction and the development of interpersonal relationships. A learning environment with higher levels of intimacy and interactivity can help meet the psychological need for motivation. We also found that when MIM was used in optional tasks, in which the students could choose whether and how they want to participate in the discussion, MIM seemed to afford a higher level of cognitive engagement, as shown by more indicators of idea exchange and creativity. MIM emerges as a promising tool for engaging students in social learning activities and fostering higher-level thinking through interaction. Our findings provide evidence to improve the instructional design of MIM-supported learning experiences and promote our theoretical understanding of student engagement.

## Data Availability

The datasets generated and analysed during the current study are not publicly available under the university IRB guidelines. Other materials are available from the corresponding author on reasonable request.
